# Tumor Restrictive Suicide Gene Therapy for Glioma Controlled by the FOS Promoter

**DOI:** 10.1371/journal.pone.0143112

**Published:** 2015-11-16

**Authors:** Jianqing Pan, Hao Wang, Xinmin Liu, Jiliang Hu, Weijian Song, Jie Luo, Shan Jiang, Fei Yan, Baojin Zhai

**Affiliations:** 1 Stroke Center, The Affiliated Shenzhen Nanshan Hospital, Guangdong Medical College, Shenzhen, Guangdong, China; 2 Department of Neurology, The Affiliated Shenzhen Nanshan Hospital, Shenzhen University, Shenzhen, Guangdong, China; 3 Department of Neurosurgery, Shenzhen People's Hospital, Jinan University, Shenzhen, Guangdong, China; 4 Institute for Advanced Study, Shenzhen University, Shenzhen, Guangdong, China; 5 Paul C. Lauterbur Research Center for Biomedical Imaging, Institute of Biomedical and Health Engineering, Shenzhen Institutes of Advanced Technology, Chinese Academy of Sciences, Shenzhen Guangdong, China; Kyung Hee University, REPUBLIC OF KOREA

## Abstract

Effective suicide gene delivery and expression are crucial to achieving successful effects in gene therapy. An ideal tumor-specific promoter expresses therapeutic genes in tumor cells with minimal normal tissue expression. We compared the activity of the FOS (FBJ murine osteosarcoma viral oncogene homolog) promoter with five alternative tumor-specific promoters in glioma cells and non-malignant astrocytes. The FOS promoter caused significantly higher transcriptional activity in glioma cell lines than all alternative promoters with the exception of CMV. The FOS promoter showed 13.9%, 32.4%, and 70.8% of the transcriptional activity of CMV in three glioma cell lines (U87, U251, and U373). Importantly, however, the FOS promoter showed only 1.6% of the transcriptional activity of CMV in normal astrocytes. We also tested the biologic activity of recombinant adenovirus containing the suicide gene herpes simplex virus thymidine kinase (HSV-tk) driven by the FOS promoter, including selective killing efficacy *in vitro* and tumor inhibition rate *in vivo*. Adenoviral-mediated delivery of the HSV-tk gene controlled by the FOS promoter conferred a cytotoxic effect on human glioma cells *in vitro* and *in vivo*. This study suggests that use of the FOS-tk adenovirus system is a promising strategy for glioma-specific gene therapy but still much left for improvement.

## Introduction

Suicide genes are widely used in cancer gene therapy to confer a cytotoxic effect to tumor cells. The herpes simplex virus thymidine kinase gene combined with ganciclovir (HSV-tk/GCV) suicide gene therapy system is considered to be one of the most promising therapeutic strategies for malignant gliomas [1]. The HSV-tk suicide gene encodes an enzyme that is able to phosphorylate antiviral prodrugs such as ganciclovir (GCV) or acyclovir (ACV) into toxic forms that lead to cell death. In addition to killing transduced tumor cells, an attractive feature of this system is a bystander effect in which non-transduced tumor cells are also killed. Since its inception, HSV-tk suicide gene therapy has been employed in many experimental settings, including treatment of glioma, ovarian cancer, malignant melanoma and other tumors [2–4].

Efficient expression of suicide genes is crucial to a successful gene therapy system. The CMV promoter has been widely used for this purpose, as it is one of the strongest promoters in mammalian cells [5]. The expression of HSV-tk driven by the CMV promoter induced cytotoxicity in glioma cells in presence of ganciclovir. However, the CMV promoter induces target gene expression in both normal and cancer cells resulting in cytotoxicity in both glioma tissue and non-malignant astrocytes. An attractive strategy to solve this problem, therefore, is the use of a tumor-specific promoter.

FOS is an important component of Activating Protein 1 (AP-1), which is a dimeric transcription factor containing members of FOS, JUN, ATF and MAF proteins [6]. AP-1 converts extracellular signals into changes in the expression of specific target genes which harbor AP-1 binding sites in their promoter or enhancer [7]. As an AP-1 component, FOS has been implicated in the regulation of various cellular processes such as enhanced proliferation, apoptosis, and tumor metastasis [8].

A number of studies have investigated the oncogenic functions of FOS and found its target genes to be critical for tumorigenesis, responsible for invasive growth of tumor cells and inhibition of tumor suppressor activity [9, 10]. In breast cancer, increased Fos protein expression was associated with a poor prognosis [11]. An immunohistochemical study of more than 600 patients with gastric carcinoma showed that loss of FOS expression was associated with adverse outcome [12]. The expression of FOS protein was found to be increased in other tumor types including cervical cancer, squamous cell cancer, and endometrial cancer [13–15]. mRNA analysis shows increased FOS levels in high grade glioma tissue compared with low grade glioma and normal brain tissue [16].

The activity of the human FOS and CMV promoters has been compared in various mammalian cell lines. The FOS promoter was 1.8–5.6-fold stronger than the CMV promoter in BHK-A, BHK-B, CHO-DHFR(-), and mouse NIH-3T3 cells, and less effective in mouse myeloma or human 293 cells [17]. However, the transcriptional activity and expression selectivity of the FOS promoter in human glioma cell lines remains untested. Furthermore FOS promoter hasn't been used to develop transcriptionally targeted gene therapy. In the present study, we compared FOS promoter activity with four tumor-specific promoters in human glioma cell lines and astrocytes, and tested the biologic activity of recombinant adenovirus containing suicide genes driven by FOS promoter, including selective killing efficacy *in vitro* and tumor inhibition rate *in vivo*.

## Materials and Methods

### Plasmid construction

DNA vectors were constructed using standard molecular biology techniques, followed by restriction analysis and sequencing of subcloned regions. A 775-bp fragment of the human FOS promoter (from −682 to +92 bp relative to the transcriptional start site) was amplified by PCR from human genomic DNA using forward (5′-CTACTGGTACCGCAGGAACAGTGCTAGTATTGCTC-3′) and reverse (5′-CTCGTAAGCTTGGCTCAGTCTTGGCTTCTCAG-3′) primers [17]. The FOS promoter was digested with Kpn l and HindIII, and cloned upstream of the luciferase gene into similarly cut pGL4-Basic plasmid (Promega, Madison, WI, USA) to generate pGL4-FOS.

A 1568-bp fragment of the human cyclo-oxygenase 2 (COX2) promoter (from −1432 to +59 bp relative to the transcriptional start site) was amplified by PCR from the plasmid pDRIVE5s-hCOX-2 (InvivoGen, San Diego, CA, USA) using forward (5′-CTACTGGTACCCACTAGTT GAGGTACCTGGTGTAG-3′) and reverse (5′-CTCGTAAGCTTGAC AGCGGCGGGCAG-3′) primers. The COX-2 promoter was digested with Kpn l and HindIII, and cloned upstream of the luciferase gene into similarly cut pGL4-Basic plasmid to generate pGL4-COX2.

A 399-bp fragment of the human E2F-1 promoter (GenBank accession no. S74230) was amplified by PCR from plasmid pDRIVE5s-hE2F-1 using forward (5′-CTACTGGTACCGTGTCCCCAC GCCTCCAG-3′) and reverse (5′-CTCGTAAGCTTGACGCTCACGGC CCG-3′) primers. The E2F1 promoter was digested with Kpn l and HindIII, and cloned upstream of the luciferase gene into similarly cut pGL4-Basic plasmid to generate pGL4-E2F1.

A 278-bp fragment of the human Survivin promoter was amplified by PCR from plasmid pDRIVE02-Survivin (InvivoGen, San Diego, CA, USA) using the forward (5′-CTACTGGTACCCCCACTAGTTCTTT GAAAGCAGTCG-3′) and reverse (5′-CTCGTAAGCTTATGCCGCCGC CGCC-3′) primers. The Survivin promoter was digested with Kpn l and HindIII, and cloned upstream of the luciferase gene into similarly cut pGL4-Basic plasmid to generate pGL4-Survivin.

A 1672-bp fragment of the human TERT promoter (GenBank accession no. AF098956) was amplified by PCR from human genomic DNA using forward (5′-CTACTGGTACCATCATCAGCTTTTCAA AGACACACT-3′) and reverse (5′-CTCGTAAGCTTGCGCTGCCTGA AACTCGC-3′) primers. The TERT promoter was digested with Kpn l and HindIII, and cloned upstream of the luciferase gene into similarly cut pGL4-Basic plasmid to generate pGL4-TERT.

### Cell lines

The cell lines U87, U373, U251, and HEK293 were purchased from the American Type Culture Collection (Manassas, VA). U87 and U373 cell lines were grown in Dulbecco's modified Eagle's medium (DMEM), supplemented with 10% fetal calf serum (FCS; HyClone, Logan, UT, USA), 2 mM glutamine, 100 U/ml penicillin and 100 μg/ml streptomycin. Human astrocytes were purchased from the ScienCell Research Laboratories (Carlsbad, CA) and grown in Astrocyte Medium (ScienCell, Carlsbad, CA). All cell lines were cultured in a 37°C incubator containing 5% CO_2_.

### Transient transfections and dual-luciferase assays

For transfection, 4 × 10^3^ cells were seeded into each well of a 96-well plate and grown for 24 h. Cells were washed, placed in Opti-MEM and co-transfected using TurboFect Transfection Reagent (Thermo Scientific, Rockford, IL, USA) with the pGL4 promoter construct along with pRL-TK Renilla luciferase constructs (Promega Corp. Madison, WI, USA) as a control for transfection efficiency. Cells were harvested 48h post-transfection and luciferase assays were performed using the Dual-Luciferase Assay System (Promega Corp. Madison, WI, USA). Relative light units were determined using a Lumat LB9501 luminometer (Berthold Technologies, Bad Wildbad, Germany) for both firefly and Renilla luciferase. Firefly luciferase activity was normalized for Renilla luciferase activity.

### Mice

Four- to six-week-old female athymic nude mice were purchased from Guangdong Medical Laboratory Animal Center. Mice were housed at the Animal Facility of Shenzhen Third People's Hospital. The protocol was approved by the Committee on the Ethics of Animal Experiments of the Shenzhen Third People's Hospital (Permit Number: 2013–1206). Before tumor implantation, mice were treated with intraperitoneal injection of 1% sodium pentobarbital at a dose of 40mg/kg. Before removal of tumor, mice were subjected to euthanasia at a dose of 150mg/kg.. A total of 33 mice were used, 15 mice for measurement of luciferase activity *in vitro* (n = 5 per group), and 18 mice for xenograft animal model (n = 6 per group).

### Plasmid Preparation for Animal Injections

pGL4-cfos and pGL4-CMV plasmids were purified using the Endo-Free Mega Plasmid kit (Qiagen, Hilden, Germany) according to the manufacturer’s recommendations. 50 μg of plasmid DNA coding for firefly luciferase was mixed with 6 μl TurboFect *in vivo* Transfection Reagent and delivered into female athymic nude mice through tail-vein injections.

### Tissue Distribution of Luciferase Expression

To collect organs, mice were killed by cervical dislocation. Tissue specimens from heart, liver, spleen, kidney and lung were harvested and homogenized [18]. A luminometer (Lumat LB9507, Berthold Technologies) was used to measure the luciferase activity from each cell lysate, and the protein concentration was determined as described previously [8]. The luminescence results were reported as relative light units per milligram of protein.

### Construction and production of adenoviruses

A bicistronic vector pDC315-CMV-HSV1tk-IRES-hrGFP was constructed as follows: human herpes simplex virus type 1 thymidine kinase was amplified by PCR from pORF9-HSV1tk (InvivoGen), and cloned into the EcoRI/SalI sites of pDC315-IRES-hrGFP (Biowit Technologies, Shenzhen, China), generating pDC315-CMV-HSV1tk- IRES-hrGFP. The human FOS promoter fragment was amplified by PCR from pGL4-FOS, and cloned into the XbaI/EcoRI sites of pDC315-CMV-HSV1tk-IRES-hrGFP, generating pDC315-FOS-HSV1tk- IRES-hrGFP.

Approximately 1.5 × 10^6^ HEK293 cells were plated in 6-well plates 24 h before transfection, by which time they reached 50–70% confluency. 2 μg of pDC315-FOS-HSV1tk- IRES-hrGFP and 2 μg of pBHGlox(delta)E1,3Cre DNA (Microbix, Canada) was used for transfection of each well. A transfection mix was prepared by adding 4 μg of adenovirus plasmid DNA and 5 μl of polyfectine (Biowit Technologies, Shenzhen, China) to 250 μl of OptiMEM (Life Technologies, Carlsbad, CA) according to the manufacturer’s instructions. Following incubation at room temperature for 15–30 min, the transfection mix was added to the cells. After 6 h at 37°C, the media containing the transfection mix was removed, and 6 ml of growth medium was added. Transfected cells were monitored for GFP expression and collected 7–10 days after transfection by scraping cells off flasks and pelleting them along with any floating cells in the culture. All but 3 ml of the supernatant was removed. After three cycles of freezing in a methanol/dry ice bath and rapid thawing at 37°C, 1 ml of viral lysate was used to infect 3 × 10^6^ cells in a 75-cm^2^ flask. The efficiency of such infections could be conveniently followed with GFP. Three to four days later, viruses were harvested as described above. To generate higher titer viral stocks, packaging cells were infected at a multiplicity of infection (MOI) of 0.1–1 pfu/ml and grown for 3–4 days, at which time viruses were harvested as described above. This process was repeated 1–3 times, with a final round using a total of 5 × 10^8^ packaging cells in fifteen 75-cm^2^ flasks and an MOI of 1–5 pfu/ml. After 3–5 days, 50% lysis was observed, and the resultant viruses were purified by ViraBind™ Adenovirus Purification Kit (Cell Biolabs); final yields were generally 10^11^ pfu/ml.

### Cytotoxicity Assay *in vitro*

The cytotoxicity of adenovirus to glioma cells was evaluated using a colorimetric Cell Counting Kit-8 (CCK-8) assay (Dojindo, Kumamoto, Japan). Briefly, 1 × 10^4^ glioma cells were placed in 96-well plates and grown overnight. Ad-CMV-TK or Ad-cfos-TK was applied at 100 MOI or 10 MOI. Cells were incubated at 37°C for 8 h followed by changing media containing various concentrations (0.01, 0.1, 1, 10, 100 and 1000 μmol/L) of GCV (Sigma–Aldrich, St. Louis, MO, USA). The cell cultures were replenished every day with fresh medium containing GCV for the next two days. At 24 h after the last replenished cultures, the percentage of surviving cells was calculated by the CCK-8 assay. The optical density was then determined at 450 nm using a microplate reader.

### *In vivo* studies of glioma xenograft tumor models in nude mice

U373 cells were injected subcutaneously into female athymic nu/nu mice. Each animal received approximately 1 × 10^7^ cells in the left flank. When the tumor volume reached approximately 500 mm^3^, mice were treated with intratumoral injections of Ad-FOS-TK, Ad-CMV-TK (both 5×10^9^ pfu in 50 μl) or PBS every other day for a total of three doses. Each group has six mice. Starting 24 h after the initial dose of adenovirus, the mice were dosed intraperitoneally with ganciclovir (100 mg/kg) daily for a total of 14 days. Mice were monitored for tumor size and body weight once every 3 days, and the tumor volume was calculated according to the following formula: (length × width^2^)/2. We found if the animal experiments last 25 days, all the volume of tumor will be not more than 2000 mm^3^. Therefore we stopped animal experiment on the 25th day. The tumors were removed after euthanasia on the 25th day, photographed, and weighed. In order to evaluating the difference of necrotic tumor between each group, we randomly chosed 10 high power field per group and used ImageJ software to calculate the percent of viable tumor.

### Statistical analysis

All in vitro experiments were performed in triplicate. Measurement data were presented as mean ± SD. The differences between means were tested by nonparametric Mann–Whitney U test or Bonferroni’s multiple comparison t-test. The threshold for significance was *p* < 0.05. For the mouse experiments, tumor volume data were summarized using mean ± SD at baseline and multiple subsequent time points for each group of mice. Changes in tumor volume at each time point were calculated and compared between groups using t-test.

## Results

### Analysis of the cfos promoter activity *in vitro*

**Fig 1:**
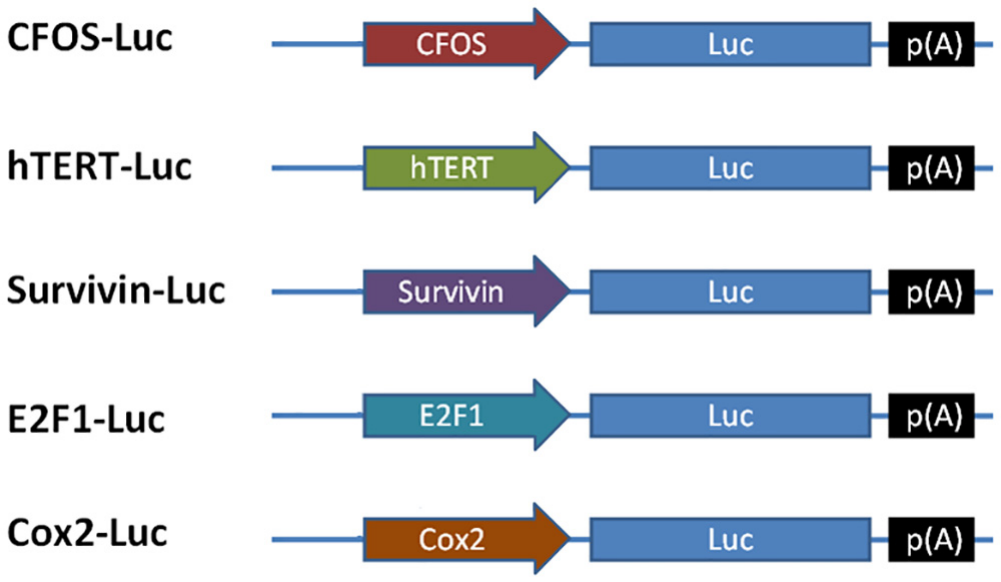
Schematic of the promoter-driven luciferase reporter plasmids.

**Fig 2:**
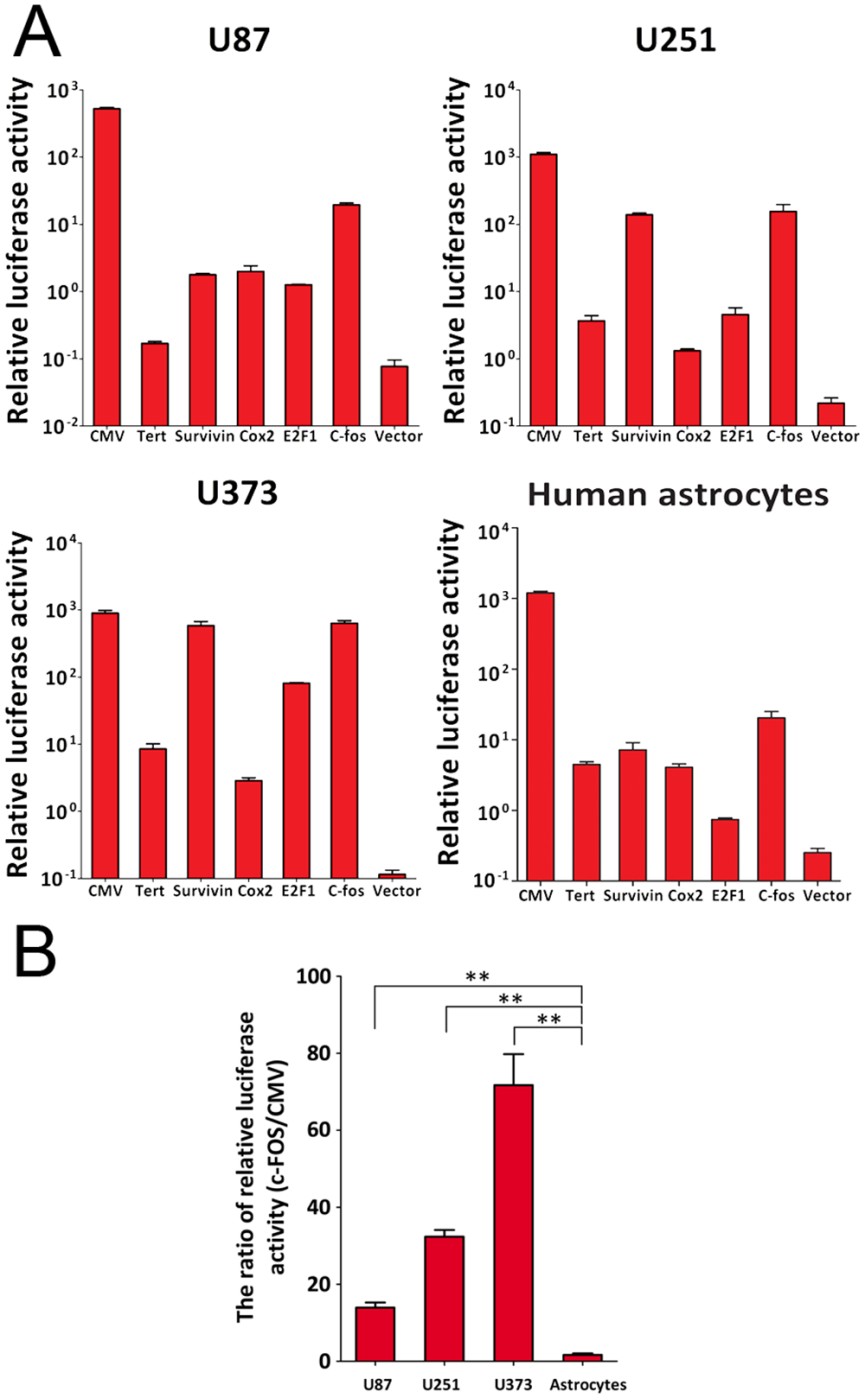
High Tumor-Specific Transcriptional Activity of the FOS Promoter. Three glioma cell lines (U87, U251, U373), and normal astrocytes cells were transiently co-transfected with plasmid DNA indicated and pRL-TK, the internal control. At 48 h following transfection, dual luciferase ratio was measured as the ratio of luciferase normalized to the Renilla luciferase internal control. A) Characterization of the plasmids in transiently transfected glioma cells and astrocytes. Relative luciferase activity is plotted on a logarithmic scale on the y-axis. Error bars are SEM. Plasmids used for transfections are noted below the x-axis. B) The ratio of relative luciferase activity is plotted on a percentage scale on the y-axis. Error bars are SEM. The data are means of multiple independent experiments. ** denotes a *p* value of < 0.01 indicating a significant difference compared with the control cell group.

To analyze the relative activity of the FOS promoter *in vitro*, cell lines were infected with pGL4-FOS, pGL4-TERT, pGL4-Survivin, pGL4-E2F1, pGL4-Cox2 or pGL4-CMV, in which the expression of the luciferase reporter gene was driven by the FOS, TERT, Survivin, E2F1, Cox2 or the CMV promoter, respectively (Fig 1). The relative luciferase activities of the tumor-specific promoters in three glioma cell lines after transfected 48h are shown in Fig 2A and Table 1. The relative luciferase activity of three glioma cell lines (U87, U251, and U373) transfected with pGL4-FOS vector was 13.9%, 32.4%, and 70.8% relative to cell lines transfected with pGL4-CMV vector (positive control; Fig 2B). Compared with the TERT, Survivin, Cox2 and E2F1 promoters, the transcriptional activity of the FOS promoter was higher in all three glioma cell lines.. Importantly, the relative luciferase activity of the FOS vector in normal astrocytes was markedly reduced at only only 1.6% of that of a CMV-driven vector in the same cells. These data indicate that the FOS promoter may be a viable alternative to CMV that is significantly less likely to cause off-target effects.

**Table 1:** The relative luciferase activities of three glioma cell lines (U87, U251, and U373) and astrocytes transfected with tumor-specific promoter vectors.

	U87		U251		U373		Asrocytes	
	Average	SD	Average	SD	Average	SD	Average	SD
**CMV**	524.68	23.9	1098.20	71.20	897.71	86.39	1201.22	144.15
**E2F1**	1.27	0.01	4.57	1.17	81.44	1.39	0.75	0.07
**FOS**	19.57	1.22	155.33	41.67	635.8	57.85	15.78	2.55
**COX2**	2.00	0.41	1.33	0.08	2.87	0.29	4.09	1.01
**TERT**	0.17	0.01	3.69	0.71	8.55	1.58	4.48	0.91
**SURVIVIN**	1.78	0.07	139.74	7.86	586.63	85.66	8.41	3.18

### Analysis of the FOS promoter activity in major organs

**Fig 3:**
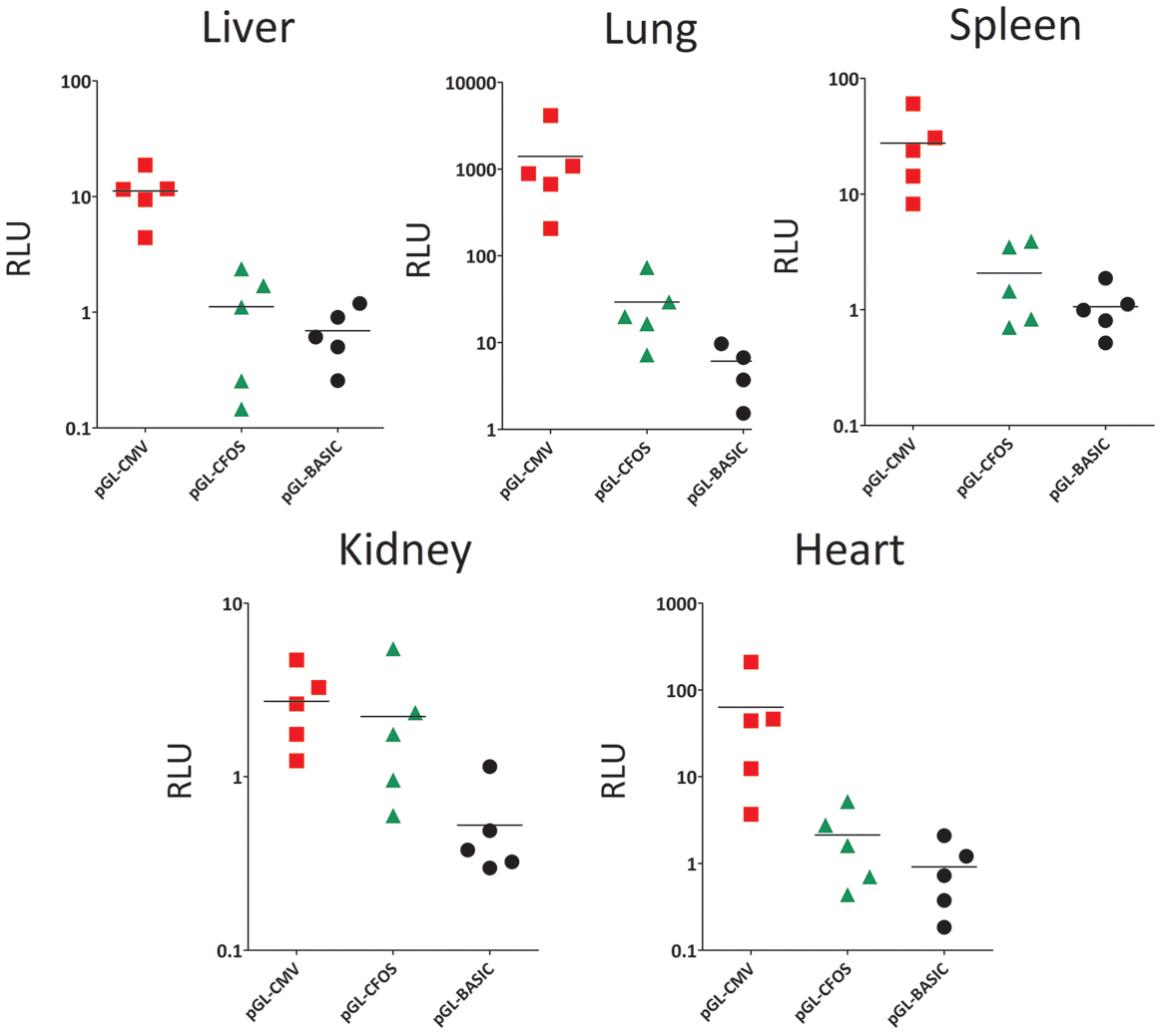
Distribution of luciferase activity in mouse major organs. Luciferase activity was measured *in vivo* in five major organs 48 h after intravenous administration of the FOS or CMV promoter-driven luciferase expression viruses. pGL4-CMV, red block; pGL4-CFOS, green triangle; pGL4-BASIC, black dot. The mean value of five samples is shown.

To measure the organ-specific activity of the FOS promoter in the mouse, pGL4-CMV and pGL4-FOS were administered intravenously. The major organs, including liver, lungs, spleen, kidneys, and heart, were harvested after 48 h, and luciferase activity was determined (Fig 3). The CMV promoter activity was relatively high in the lung and heart, and low in the kidney. The FOS promoter activity was within the same order of magnitude in all organs except lung. In the lung, the mean luciferase activity with pGL4-FOS (29.24 RLU/mg protein, n = 5) was 2.1% of the activity of pGL4-CMV. The activity in spleen of pGL4-FOS (2.07 RLU/mg protein, n = 5) was 7.5% of pGL4-CMV. The pGL4-FOS promoter activity in the heart was 3.4% (2.13 RLU/mg protein, n = 5) of the activity of pGL4-CMV. These results indicate that the FOS promoter has negligible background expression in the major organs.

### Analysis of the cfos-TK adenovirus system activity *in vitro*

**Fig 4:**
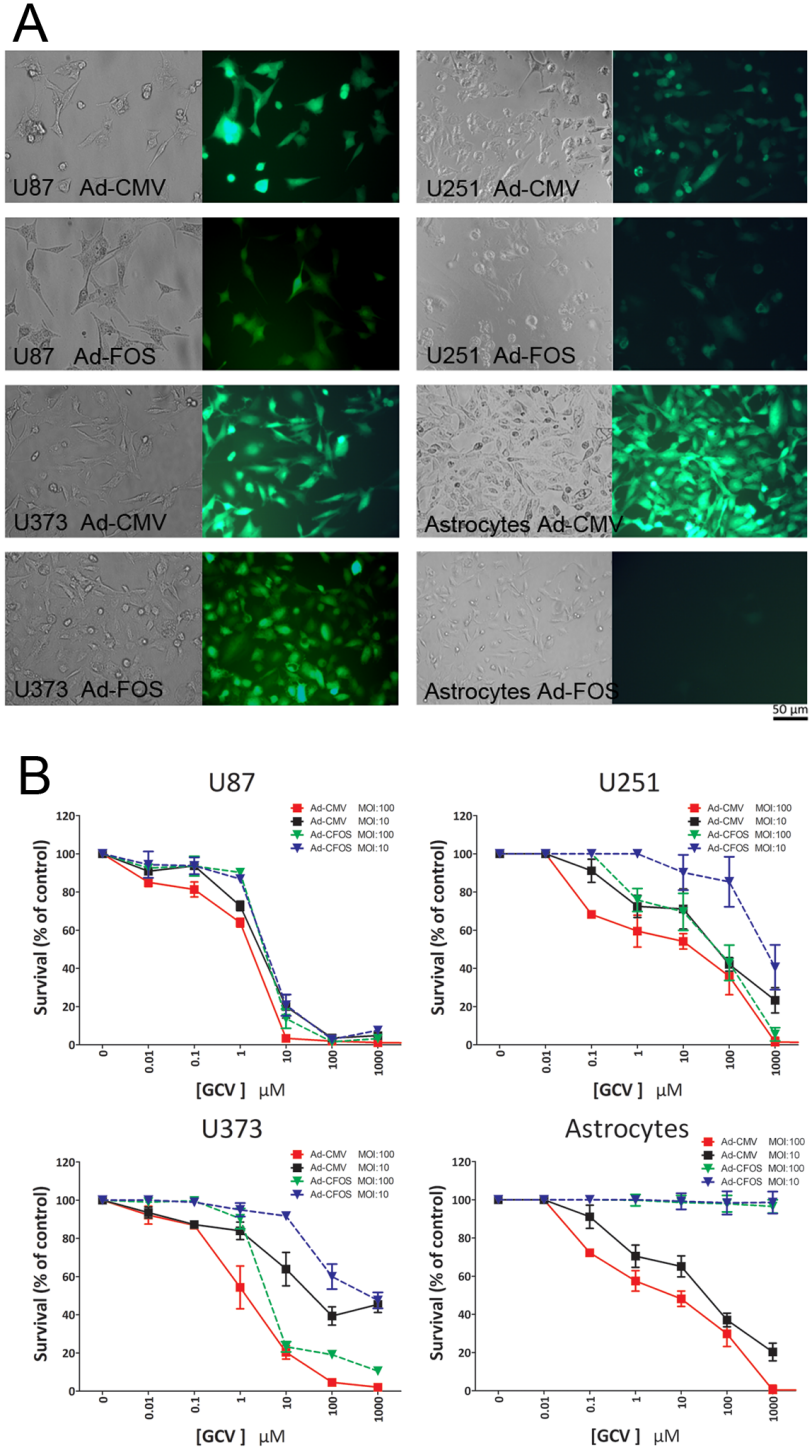
Cell killing activities of Ad-CMV-HSVtk-IRES-GFP and Ad-FOS-HSVtk- IRES-GFP in glioma cell lines and normal cells. (A) Fluorescence microscopy was used to observe U87, U251, U373 and astrocytes cells 48 h after the cells were infected with Ad-CMV-HSVtk-IRES-GFP or Ad-FOS-HSVtk-IRES-GFP at an MOI of 100 pfu/cell, original magnification 200×. (B) Three glioma cell lines and normal astrocytes were infectedwith Ad-CMV-HSVtk or Ad-FOS-HSVtk plus ganciclovir. After 48 h, the percentage of cell death was evaluated with a Cell Counting Kit-8 (CCK-8) test with the negative control set at 100%. The data shown are the means of three independent experiments.

In view of our finding that the FOS expression vector can selectively express target genes in glioma cells, we engineered a therapeutic cfos-TK adenovirus system that drives expression of HSV-tk and GFP (Ad-FOS-HSVtk-IRES- GFP; Fig 4A). In the three glioma cells lines, we compared the rate of inhibition of cell growth of Ad-FOS-HSVtk-IRES-GFP to that of Ad-CMV-HSVtk-IRES-GFP. In the presence of serially increasing GCV concentrations of 1, 10, 100, and 1000 μmol/l, the inhibition rate of Ad-FOS-HSVtk-IRES-GFP to U251 cells at MOI 100 was 24.18 ± 6.01%, 30.39 ± 9.67%, 57.07 ± 9.29%, and 94.50 ± 3.48%, respectively. In comparison the inhibition rates of Ad-CMV-HSVtk-IRES-GFP to U251 cells for the same concentrations of GCV were 36.03 ± 2.27%, 96.62±1.86%, 98.09±1.49%, and 98.84±0.60%. However, under the CMV promoter, expression of the HSV-tk gene resulted in a significant decrease in the survival of normal cells that was not observed using Ad-FOS-HSVtk-IRES-GFP (Fig 4B). The inhibition rate of Ad-FOS-HSVtk-IRES-GFP to normal astrocytes at the same four GCV concentrations was 0.73 ± 0.03%, 2.81 ± 0.13%, 4.15 ± 0.32%, 6.41 ± 0.39%, respectively. In contrast, the inhibition rates of Ad-CMV-HSVtk-IRES-GFP to normal astrocytes in the same GCV gradient were markedy higher at 43.67 ± 1.78%, 48.23 ± 1.64%, 71.52 ± 2.93%, and 99.07 ± 0.82%, respectively. Therefore, by expressing HSVtk under the FOS promoter, we demonstrated selective inhibition of cancer cells, a crucial requirement for the establishment of Ad-FOS-HSVtk as a potential therapeutic agent for glioma.

### Analysis of the FOS-TK adenovirus system activity *in vivo*

**Fig 5:**
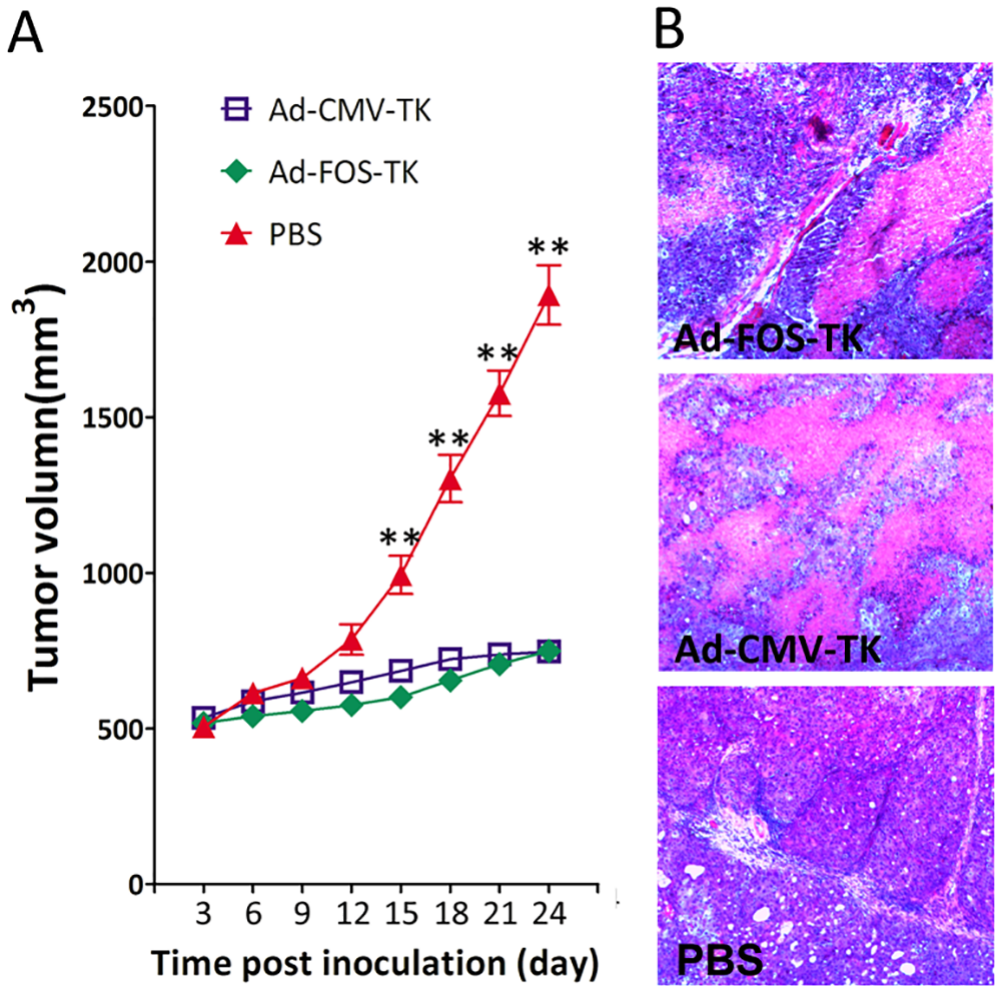
Ad-cfos-HSVtk inhibited tumor growth in a subcutaneous xenograft animal model. (A)Adenoviral gene therapy was initiated when tumors attained a volume of 500 mm^3^. Tumor volume was calculated as (length × width^2^)/2; * and ** denote *p* values of < 0.05 and < 0.01, respectively, indicating significant differences compared with the control vehicle group. (B) Representative tumor regions in routinely HE stain.

On the basis of these results, we decided to determine whether the Ad-FOS-HSVtk adenovirus system affects the growth of glioma *in vivo*. For this, we used subcutaneously implanted human U373 cells in nude mice (six mice per group). When the tumor volume reached approximately 500 mm^3^, treatment was initiated the next day by administering intratumoral injections of 5 × 10^9^ pfu /50 μl of virus or control vehicle every other day. The tumor volume in the mice treated with Ad-FOS-HSVtk-IRES-GFP was significantly smaller than that of the vehicle control group from day 15 onwards (Fig 5). 25 days after tumor inoculation, the ratio of tumor volume of Ad-FOS-HSVtk-IRES-GFP group vs vehicle control group was 39.60% (*p* < 0.0001; Fig 5). The percent of viable tumor is 85.6% in Ad-FOS-TK group, 81.8% in Ad-CMV-TK group, and 87.1% in PBS group. There was no significant difference between tumor volumes in the Ad-FOS-HSVtk-IRES-GFP group and the Ad-CMV-HSVtk-IRES-GFP group.

## Discussion

An ideal tumor-specific promoter drives tumor-specific expression of therapeutic genes with minimal normal tissue toxicity. Promoters derived from hTERT, survivin, Cox2, AFP and E2F1 gene have been used to drive tumor-specific suicide gene expression [19–22]. However, these promoters have not been strong enough to achieve therapeutic levels of transgene expression in glioma. Most tumor-specific promoters derive from tumor-associated genes. Therefore, we focused on tumor-associated genes and hypothesized that the FOS promoter may be a potential tool for glioma gene therapy.

In this study, we compared the transcriptional activities of FOS, TERT, Survivin, E2F1, Cox2 and CMV promoter in human glioma lines. Compared with TERT, Survivin, Cox2 and E2F1 promoter, the transcriptional activity of FOS promoter was higher in glioma cell lines. In addition, the FOS promoter has much lower transcriptional activity in normal tissue than CMV promoter. We constructed recombinant adenovirus vectors containing the HSV-tk gene controlled by a human FOS promoter. We found that adenoviral-mediated delivery of the HSV-tk gene controlled by the FOS promoter can confer cytotoxic effect on human glioma cells *in vitro* and *in vivo*.

The FOS promoter has been widely applied in neuroscience research. One study has shown either repeated high-frequency bursts (50Hz) or prolonged, low-frequency stimuli (5Hz) induces FOS expression [23]. Barth *et al*. generated a FosGFP transgenic mouse, in which a cFOS-GFP fusion protein was expressed downstream of a FOS promoter [24]. A study with FosGFP transgenic mice showed that an increased spontaneous activity was correlated with the FosGFP expression in cortex pyramidal neurons [25]. In keeping with these data we have also found that the cfos-promoter adenovirus system can express target genes in pyramidal neurons via an activity-dependent mechanism (data not shown). Therefore, the cfos-promoter adenovirus system needs further refinement to reduce the risk of killing excited neurons.

In conclusion, we have developed a glioma-specific gene therapy system that expresses the HSV-tk suicide gene under direct transcriptional control of the FOS promoter, and have validated the therapeutic effects of this system in glioma *in vitro* and *in vivo*. The data presented indicate that the use of FOS-TK adenovirus system is a promising strategy to deliver glioma-specific gene therapy but still much left for improvement.

## Supporting Information

S1 Table Raw data of the relative luciferase activities of three glioma cell lines (U87, U251, and U373) and astrocytes transfected with tumor- specific promoter vectors. (DOCX)

S2 Table Raw data of distribution of luciferase activity in mouse major organs. (DOCX)

S3 Table Raw data of the cell killing activities of Ad-CMV-HSVtk-IRES- GFP and Ad-FOS-HSVtk-IRES-GFP in glioma cell lines and normal cells. (DOCX)

S4 Table Tumor volumes of the subcutaneous xenograft animal model. (DOCX)
