# Supplementary material for: Tumor Restrictive Suicide Gene Therapy for Glioma Controlled by the FOS Promoter
Source: PLoS One. 2015 Nov 16;10(11):e0143112. doi: 10.1371/journal.pone.0143112 (PMC4646428; doi:10.1371/journal.pone.0143112)
Supplement: S1 Table — (DOCX) [file pone.0143112.s001.docx]

**Table S1** Raw data of the relative luciferase activities of three glioma cell lines (U87, U251, and U373) and astrocytes transfected with tumor-specific promoter vectors.

**U87 cell line**

|  |  |  | 1. pGL4-CMV | |  |  |
| --- | --- | --- | --- | --- | --- | --- |
|  |  | well-1 | well-2 | well-3 | average | SD |
|  | luciferase | 99670.51 | 91267.37 | 99853.56 |  |  |
|  | renila | 199.97 | 172.07 | 183.15 |  |  |
|  | L/R | 498.44 | 530.42 | 545.19 | 524.68 | 23.90 |
|  |  |  |  |  |  |  |
|  |  |  | 2. pGL4-E2F1 | |  |  |
|  |  | well-1 | well-2 | well-3 | average | SD |
|  | luciferase | 168.07 | 150.46 | 152.47 |  |  |
|  | renila | 130.68 | 119.19 | 121.08 |  |  |
|  | L/R | 1.29 | 1.26 | 1.26 | 1.27 | 0.01 |
|  |  |  |  |  |  |  |
|  |  |  | 3. pGL4-FOS | |  |  |
|  |  | well-1 | well-2 | well-3 | average | SD |
|  | luciferase | 2196.73 | 2393.66 | 2424.64 |  |  |
|  | renila | 120.78 | 119.56 | 118.22 |  |  |
|  | L/R | 18.19 | 20.02 | 20.51 | 19.57 | 1.22 |
|  |  |  |  |  |  |  |
|  |  |  | 4. pGL4-COX | |  |  |
|  |  | well-1 | well-2 | well-3 | average | SD |
|  | luciferase | 294.98 | 224.14 | 266.27 |  |  |
|  | renila | 118.97 | 128.00 | 150.25 |  |  |
|  | L/R | 2.48 | 1.75 | 1.77 | 2.00 | 0.41 |
|  |  |  |  |  |  |  |
|  |  |  | 5. pGL4-hTert | |  |  |
|  |  | well-1 | well-2 | well-3 | average | SD |
|  | luciferase | 19.32 | 21.37 | 23.20 |  |  |
|  | renila | 119.58 | 128.22 | 127.07 |  |  |
|  | L/R | 0.16 | 0.17 | 0.18 | 0.17 | 0.01 |
|  |  |  |  |  |  |  |
|  |  |  | 6. pGL4-suriv | |  |  |
|  |  | well-1 | well-2 | well-3 | average | SD |
|  | luciferase | 218.17 | 223.44 | 208.59 |  |  |
|  | renila | 118.90 | 123.20 | 122.73 |  |  |
|  | L/R | 1.83 | 1.81 | 1.70 | 1.78 | 0.07 |
|  |  |  |  |  |  |  |
|  |  |  | 7. pGL4-basic | |  |  |
|  |  | well-1 | well-2 | well-3 | average | SD |
|  | luciferase | 7.17 | 6.20 | 6.03 |  |  |
|  | renila | 72.61 | 88.92 | 93.97 |  |  |
|  | L/R | 0.10 | 0.07 | 0.06 | 0.08 | 0.02 |

**U251 cell line**

|  |  |  | 1. pGL4-CMV | |  |  |  |
| --- | --- | --- | --- | --- | --- | --- | --- |
|  |  | well-1 | well-2 | | well-3 | average | SD |
|  | luciferase | 190213.08 | 190352.63 | | 140712.53 |  |  |
|  | renila | 162.61 | 173.44 | | 136.97 |  |  |
|  | L/R | 1169.75 | 1097.51 | | 1027.35 | 1098.20 | 71.20 |
|  |  |  |  | |  |  |  |
|  |  |  | 2. pGL4-E2F1 | |  |  |  |
|  |  | well-1 | well-2 | | well-3 | average | SD |
|  | luciferase | 906.02 | 983.00 | | 932.95 |  |  |
|  | renila | 226.97 | 166.07 | | 245.83 |  |  |
|  | L/R | 3.99 | 5.92 | | 3.80 | 4.57 | 1.17 |
|  |  |  |  | |  |  |  |
|  |  |  | 3. pGL4-FOS | |  |  |  |
|  |  | well-1 | well-2 | | well-3 | average | SD |
|  | luciferase | 21197.86 | 15282.24 | | 19418.36 |  |  |
|  | renila | 106.24 | 130.86 | | 129.73 |  |  |
|  | L/R | 199.53 | 116.78 | | 149.68 | 155.33 | 41.67 |
|  |  |  |  | |  |  |  |
|  |  |  | 4. pGL4-COX | |  |  |  |
|  |  | well-1 | well-2 | | well-3 | average | SD |
|  | luciferase | 164.24 | 153.80 | | 167.51 |  |  |
|  | renila | 121.80 | 124.34 | | 120.49 |  |  |
|  | L/R | 1.35 | 1.24 | | 1.39 | 1.33 | 0.08 |
|  |  |  |  | |  |  |  |
|  |  |  | 5. pGL4-hTert | | |  |  |
|  |  | well-1 | well-2 | well-3 | | average | SD |
|  | luciferase | 586.83 | 680.07 | 437.92 | |  |  |
|  | renila | 143.07 | 166.14 | 153.00 | |  |  |
|  | L/R | 4.10 | 4.09 | 2.86 | | 3.69 | 0.71 |
|  |  |  |  |  | |  |  |
|  |  |  | 6. pGL4-suriv | | |  |  |
|  |  | well-1 | well-2 | well-3 | | average | SD |
|  | luciferase | 19312.27 | 19646.42 | 15155.83 | |  |  |
|  | renila | 147.80 | 136.32 | 104.93 | |  |  |
|  | L/R | 130.67 | 144.12 | 144.43 | | 139.74 | 7.86 |
|  |  |  |  |  | |  |  |
|  |  |  | 7. pGL4-basic | | |  |  |
|  |  | well-1 | well-2 | well-3 | | average | SD |
|  | luciferase | 5.41 | 5.90 | 6.61 | |  |  |
|  | renila | 23.88 | 34.22 | 25.58 | |  |  |
|  | L/R | 0.23 | 0.17 | 0.26 | | 0.22 | 0.04 |

**U373 cell line**

|  |  |  | 1. pGL4-CMV |  |  |  |
| --- | --- | --- | --- | --- | --- | --- |
|  |  | well-1 | well-2 | well-3 | average | SD |
|  | luciferase | 646027.53 | 471161.80 | 386244.61 |  |  |
|  | renila | 667.83 | 509.51 | 482.19 |  |  |
|  | L/R | 967.35 | 924.74 | 801.03 | 897.71 | 86.39 |
|  |  |  |  |  |  |  |
|  |  |  | 2. pGL4-E2F1 | |  |  |
|  |  | well-1 | well-2 | well-3 | average | SD |
|  | luciferase | 28480.95 | 15020.59 | 10656.10 |  |  |
|  | renila | 356.68 | 182.14 | 129.97 |  |  |
|  | L/R | 79.85 | 82.47 | 81.99 | 81.44 | 1.39 |
|  |  |  |  |  |  |  |
|  |  |  | 3. pGL4-FOS |  |  |  |
|  |  | well-1 | well-2 | well-3 | average | SD |
|  | luciferase | 213842.75 | 165086.71 | 229359.27 |  |  |
|  | renila | 364.86 | 265.41 | 327.98 |  |  |
|  | L/R | 586.09 | 622.01 | 699.30 | 635.80 | 57.85 |
|  |  |  |  |  |  |  |
|  |  |  | 4. pGL4-COX |  |  |  |
|  |  | well-1 | well-2 | well-3 | average | SD |
|  | luciferase | 636.71 | 888.71 | 682.10 |  |  |
|  | renila | 246.54 | 281.07 | 237.19 |  |  |
|  | L/R | 2.58 | 3.16 | 2.88 | 2.87 | 0.29 |
|  |  |  |  |  |  |  |
|  |  |  | 5. pGL4-hTert | |  |  |
|  |  | well-1 | well-2 | well-3 | average | SD |
|  | luciferase | 3411.86 | 3168.17 | 3154.32 |  |  |
|  | renila | 419.15 | 439.31 | 306.39 |  |  |
|  | L/R | 8.14 | 7.21 | 10.30 | 8.55 | 1.58 |
|  |  |  |  |  |  |  |
|  |  |  | 6. pGL4-suriv | |  |  |
|  |  | well-1 | well-2 | well-3 | average | SD |
|  | luciferase | 81217.49 | 91321.53 | 94834.44 |  |  |
|  | renila | 136.08 | 136.97 | 191.07 |  |  |
|  | L/R | 596.82 | 666.75 | 496.34 | 586.63 | 85.66 |
|  |  |  |  |  |  |  |
|  |  |  | 7. pGL4-basic | |  |  |
|  |  | well-1 | well-2 | well-3 | average | SD |
|  | luciferase | 6.22 | 7.27 | 7.36 |  |  |
|  | renila | 46.27 | 72.29 | 65.88 |  |  |
|  | L/R | 0.13 | 0.10 | 0.11 | 0.12 | 0.02 |

**Astrocytes**

|  |  |  |  | 1.pGL4-CMV | |  |  |  |
| --- | --- | --- | --- | --- | --- | --- | --- | --- |
|  |  | well-1 | well-2 | well-3 | well-4 | well-5 | average | SD |
|  | luciferase | 63507.98 | 55868.92 | 55060.64 | 33274.95 | 120904.73 |  |  |
|  | renila | 50.07 | 48.25 | 53.78 | 23.64 | 105.25 |  |  |
|  | L/R | 1268.44 | 1157.80 | 1023.82 | 1407.33 | 1148.69 | 1201.22 | 144.15 |
|  |  |  |  |  |  |  |  |  |
|  |  |  |  | 2.pGL4-E2F1 | |  |  |  |
|  |  | well-1 | well-2 | well-3 | well-4 | well-5 | average | SD |
|  | luciferase | 22.15 | 30.17 | 27.36 | 32.15 | 40.56 |  |  |
|  | renila | 27.71 | 37.00 | 42.90 | 40.68 | 58.36 |  |  |
|  | L/R | 0.80 | 0.82 | 0.64 | 0.79 | 0.70 | 0.75 | 0.08 |
|  |  |  |  |  |  |  |  |  |
|  |  |  |  | 3.pGL4-FOS | |  |  |  |
|  |  | well-1 | well-2 | well-3 | well-4 | well-5 | average | SD |
|  | luciferase | 956.11 | 1220.92 | 624.26 | 629.06 | 901.70 |  |  |
|  | renila | 66.56 | 68.86 | 36.14 | 35.75 | 75.42 |  |  |
|  | L/R | 14.36 | 17.73 | 17.28 | 17.60 | 11.96 | 15.78 | 2.55 |
|  |  |  |  |  |  |  |  |  |
|  |  |  |  | 4.PGL4-COX | |  |  |  |
|  |  | well-1 | well-2 | well-3 | well-4 | well-5 | average | SD |
|  | luciferase | 147.36 | 146.97 | 246.03 | 152.14 | 103.05 |  |  |
|  | renila | 36.58 | 32.25 | 45.80 | 38.83 | 39.42 |  |  |
|  | L/R | 4.03 | 4.56 | 5.37 | 3.92 | 2.61 | 4.10 | 1.01 |
|  |  |  |  |  |  |  |  |  |
|  |  |  |  | 5.PGL4-hTert | |  |  |  |
|  |  | well-1 | well-2 | well-3 | well-4 | well-5 | average | SD |
|  | luciferase | 418.71 | 167.61 | 130.25 | 118.19 | 117.66 |  |  |
|  | renila | 119.59 | 48.17 | 25.37 | 23.68 | 22.29 |  |  |
|  | L/R | 3.50 | 3.48 | 5.13 | 4.99 | 5.28 | 4.48 | 0.91 |
|  |  |  |  |  |  |  |  |  |
|  |  |  |  | 6.PGL4-suriv | |  |  |  |
|  |  | well-1 | well-2 | well-3 | well-4 | well-5 | average | SD |
|  | luciferase | 760.00 | 128.73 | 339.61 | 420.36 | 137.05 |  |  |
|  | renila | 55.42 | 36.41 | 58.68 | 47.14 | 33.53 |  |  |
|  | L/R | 13.71 | 3.54 | 5.79 | 8.92 | 4.09 | 7.21 | 4.20 |
|  |  |  |  |  |  |  |  |  |
|  |  |  |  | 7.PGL4-basic | |  |  |  |
|  |  | well-1 | well-2 | well-3 | well-4 | well-5 | average | SD |
|  | luciferase | 16.56 | 16.61 | 15.73 | 14.61 | 14.37 |  |  |
|  | renila | 64.53 | 65.02 | 108.25 | 63.54 | 38.29 |  |  |
|  | L/R | 0.26 | 0.26 | 0.15 | 0.23 | 0.38 | 0.25 | 0.08 |
