# Supplementary material for: Tumor Restrictive Suicide Gene Therapy for Glioma Controlled by the FOS Promoter
Source: PLoS One. 2015 Nov 16;10(11):e0143112. doi: 10.1371/journal.pone.0143112 (PMC4646428; doi:10.1371/journal.pone.0143112)
Supplement: S2 Table — (DOCX) [file pone.0143112.s002.docx]

**Table S2** Raw data of distribution of luciferase activity in mouse major organs.

Luciferase activity was measured in vivo in five major organs 48 h after intravenous administration of the FOS or CMV promoter-driven luciferase expression vectors.

|  | **HEART** |  |
| --- | --- | --- |
| pGL-CMV | pGL-FOS | pGL-BASIC |
| 12.407 | 0.703 | 0.375 |
| 46.450 | 2.754 | 0.184 |
| 3.695 | 1.609 | 2.092 |
| 210.160 | 0.436 | 1.211 |
| 44.346 | 5.154 | 0.725 |
|  |  |  |
|  | **LIVER** |  |
| pGL-CMV | pGL-FOS | pGL-BASIC |
| 9.407 | 0.145 | 0.903 |
| 11.569 | 2.366 | 0.256 |
| 4.410 | 1.103 | 1.188 |
| 11.699 | 0.254 | 0.500 |
| 18.725 | 1.693 | 0.609 |
|  |  |  |
|  | **KIDNEY** |  |
| pGL-CMV | pGL-FOS | pGL-BASIC |
| 1.760 | 0.956 | 0.378 |
| 1.238 | 5.466 | 1.143 |
| 2.632 | 0.596 | 0.323 |
| 3.278 | 1.755 | 0.488 |
| 4.714 | 2.340 | 0.298 |
|  |  |  |
|  | **LUNG** |  |
| pGL-CMV | pGL-FOS | pGL-BASIC |
| 1084.901 | 16.518 | 6.714 |
| 887.783 | 19.864 | 1.531 |
| 206.933 | 29.304 | 3.706 |
| 669.406 | 7.171 | 8.917 |
| 4147.463 | 73.339 | 9.668 |
|  |  |  |
|  | **SPLEEN** |  |
| pGL-CMV | pGL-FOS | pGL-BASIC |
| 23.912 | 1.448 | 1.115 |
| 14.359 | 0.702 | 0.804 |
| 8.223 | 3.478 | 0.516 |
| 30.664 | 0.830 | 1.870 |
| 60.488 | 3.900 | 0.992 |
