# Supplementary material for: Tumor Restrictive Suicide Gene Therapy for Glioma Controlled by the FOS Promoter
Source: PLoS One. 2015 Nov 16;10(11):e0143112. doi: 10.1371/journal.pone.0143112 (PMC4646428; doi:10.1371/journal.pone.0143112)
Supplement: S3 Table — (DOCX) [file pone.0143112.s003.docx]

**Table S3** Raw data of the cell killing activities of Ad-CMV-HSVtk-IRES-GFP and Ad-FOS-HSVtk- IRES-GFP in glioma cell lines and normal cells.

U251

|  | Ad-CMV | | | | Ad-FOS | | | |
| --- | --- | --- | --- | --- | --- | --- | --- | --- |
| GCV concentrations（μmol/L） | MOI:100 | | MOI:10 | | MOI:100 | | MOI:10 | |
|  | average | SD | average | SD | average | SD | average | SD |
| 0 | 0.00 | 0.00 | 0.00 | 0.00 | 0.00 | 0.00 | 0.00 | 0.00 |
| 0.01 | 0.00 | 0.00 | 0.00 | 0.00 | 0.00 | 0.00 | 0.00 | 0.00 |
| 0.1 | 31.75 | 1.81 | 8.90 | 6.06 | 0.00 | 0.00 | 0.00 | 0.00 |
| 1 | 40.50 | 8.37 | 27.53 | 5.81 | 24.18 | 6.01 | 0.00 | 0.00 |
| 10 | 45.85 | 4.05 | 28.88 | 10.51 | 30.39 | 9.67 | 9.79 | 9.22 |
| 100 | 64.18 | 9.61 | 57.92 | 3.56 | 57.07 | 9.29 | 14.65 | 13.08 |
| 1000 | 98.57 | 2.48 | 76.70 | 6.63 | 94.50 | 3.48 | 59.44 | 11.72 |

U87

|  | Ad-CMV | | | | Ad-FOS | | | |
| --- | --- | --- | --- | --- | --- | --- | --- | --- |
| GCV concentrations（μmol/L） | MOI:100 | | MOI:10 | | MOI:100 | | MOI:10 | |
|  | average | SD | average | SD | average | SD | average | SD |
| 0 | 0.00 | 0.00 | 0.00 | 0.00 | 0.00 | 0.00 | 0.00 | 0.00 |
| 0.01 | 14.94 | 2.21 | 9.14 | 3.56 | 7.45 | 2.34 | 5.68 | 6.92 |
| 0.1 | 18.67 | 3.92 | 6.31 | 4.42 | 6.44 | 5.19 | 6.31 | 4.42 |
| 1 | 36.03 | 2.27 | 27.29 | 2.48 | 2.78 | 2.24 | 13.22 | 0.72 |
| 10 | 96.62 | 1.86 | 79.94 | 6.06 | 86.32 | 5.06 | 79.25 | 5.56 |
| 100 | 98.09 | 1.49 | 96.46 | 1.44 | 98.48 | 0.79 | 97.18 | 2.08 |
| 1000 | 98.85 | 0.60 | 95.19 | 1.79 | 96.76 | 0.93 | 92.47 | 2.21 |

U373

|  | Ad-CMV | | | | Ad-FOS | | | |
| --- | --- | --- | --- | --- | --- | --- | --- | --- |
| GCV concentrations（μmol/L） | MOI:100 | | MOI:10 | | MOI:100 | | MOI:10 | |
|  | average | SD | average | SD | average | SD | average | SD |
| 0 | 0.00 | 0.00 | 0.00 | 0.00 | 0.00 | 0.00 | 0.00 | 0.00 |
| 0.01 | 7.84 | 4.69 | 6.50 | 2.86 | 0.99 | 0.00 | 0.00 | 0.00 |
| 0.1 | 13.14 | 1.04 | 12.78 | 0.16 | 0.35 | 1.44 | 1.13 | 1.28 |
| 1 | 45.69 | 11.18 | 16.07 | 4.59 | 9.64 | 5.33 | 5.05 | 3.52 |
| 10 | 79.66 | 3.53 | 36.09 | 8.74 | 76.77 | 2.68 | 8.33 | 1.59 |
| 100 | 95.41 | 1.22 | 60.63 | 4.78 | 80.93 | 1.73 | 40.01 | 6.56 |
| 1000 | 97.95 | 1.00 | 54.63 | 4.19 | 89.51 | 1.08 | 52.47 | 4.19 |

Astrocytes

|  | Ad-CMV | | | | Ad-FOS | | | |
| --- | --- | --- | --- | --- | --- | --- | --- | --- |
| GCV concentrations（μmol/L） | MOI:100 | | MOI:10 | | MOI:100 | | MOI:10 | |
|  | average | SD | average | SD | average | SD | average | SD |
| 0 | 0.00 | 0.00 | 0.00 | 0.00 | 0.00 | 0.00 | 0.00 | 0.00 |
| 0.01 | 0.00 | 0.00 | 0.00 | 0.00 | 0.00 | 0.00 | 0.00 | 0.00 |
| 0.1 | 25.49 | 2.53 | 8.90 | 6.06 | 0.00 | 0.00 | 0.00 | 0.00 |
| 1 | 43.67 | 1.78 | 26.53 | 6.81 | 0.73 | 0.03 | 0.00 | 0.00 |
| 10 | 48.23 | 1.64 | 34.71 | 3.44 | 2.81 | 0.13 | 0.69 | 0.11 |
| 100 | 71.52 | 2.93 | 60.92 | 3.96 | 4.15 | 0.32 | 0.76 | 0.07 |
| 1000 | 99.07 | 0.82 | 76.33 | 4.79 | 6.41 | 0.39 | 0.23 | 0.04 |
