# Supplementary material for: Tumor Restrictive Suicide Gene Therapy for Glioma Controlled by the FOS Promoter
Source: PLoS One. 2015 Nov 16;10(11):e0143112. doi: 10.1371/journal.pone.0143112 (PMC4646428; doi:10.1371/journal.pone.0143112)
Supplement: S4 Table — (DOCX) [file pone.0143112.s004.docx]

**Table S4** Tumor volumes of the subcutaneous xenograft animal model.

|  | **Ad-CMV-TK** | | | | | | **Ad-FOS-TK** | | | | | | **PBS** | | | | | |
| --- | --- | --- | --- | --- | --- | --- | --- | --- | --- | --- | --- | --- | --- | --- | --- | --- | --- | --- |
|  | **mice-1** | **mice-2** | **mice-3** | **mice-4** | **mice-5** | **mice-6** | **mice-7** | **mice-8** | **mice-9** | **mice-10** | **mice-11** | **mice-12** | **mice-13** | **mice-14** | **mice-15** | **mice-16** | **mice-17** | **mice-18** |
| **day3** | 510 | 526 | 489 | 552 | 587 | 544 | 530 | 510 | 450 | 550 | 517 | 553 | 490 | 516 | 458 | 534 | 503 | 535 |
| **day6** | 560 | 573 | 571 | 585 | 627 | 612 | 552 | 538 | 457 | 579 | 541 | 573 | 620 | 637 | 673 | 581 | 596 | 578 |
| **day9** | 574 | 586 | 624 | 632 | 639 | 639 | 573 | 553 | 468 | 604 | 552 | 589 | 657 | 735 | 694 | 612 | 645 | 634 |
| **day12** | 596 | 610 | 658 | 672 | 679 | 678 | 580 | 561 | 516 | 620 | 573 | 605 | 730 | 806 | 716 | 635 | 673 | 659 |
| **day15** | 615 | 635 | 703 | 745 | 724 | 689 | 596 | 578 | 530 | 647 | 625 | 634 | 917 | 1057 | 956 | 765 | 1204 | 1062 |
| **day18** | 668 | 719 | 723 | 762 | 732 | 735 | 630 | 628 | 673 | 668 | 674 | 657 | 1450 | 1369 | 1168 | 993 | 1475 | 1363 |
| **day21** | 743 | 724 | 758 | 735 | 706 | 761 | 715 | 650 | 798 | 704 | 684 | 692 | 1635 | 1694 | 1519 | 1243 | 1734 | 1635 |
| **day24** | 763 | 753 | 783 | 693 | 708 | 783 | 751 | 721 | 859 | 736 | 693 | 738 | 1975 | 2210 | 1690 | 1576 | 1854 | 2053 |
